# Probiotics against airway allergy: host factors to consider

**DOI:** 10.1242/dmm.034314

**Published:** 2018-07-20

**Authors:** Irina Spacova, Jan L. Ceuppens, Sven F. Seys, Mariya I. Petrova, Sarah Lebeer

**Affiliations:** 1 Research Group Environmental Ecology and Applied Microbiology, Department of Bioscience Engineering, University of Antwerp, 2020 Antwerp, Belgium; 2 Centre of Microbial and Plant Genetics, Department of Microbial and Molecular Systems (M²S), KU Leuven, Belgium; 3 Laboratory of Clinical Immunology, Department of Microbiology and Immunology, KU Leuven, 3000 Leuven, Belgium

**Keywords:** Allergic asthma, Allergy, *Lactobacillus*, Microbiota, Mouse model, Probiotic

## Abstract

The worldwide prevalence of allergic diseases has drastically increased in the past decades. Recent studies underline the importance of microbial exposure for the development of a balanced immune system. Consequently, probiotic bacteria are emerging as a safe and natural strategy for allergy prevention and treatment. However, clinical probiotic intervention studies have so far yielded conflicting results. There is increasing awareness about the importance of host-associated factors that determine whether an individual will respond to a specific probiotic treatment, and it is therefore crucial to promote a knowledge-based instead of an empirical selection of promising probiotic strains and their administration regimen.

In this Review, we summarize the insights from animal model studies of allergic disease, which reveal how host-related factors – such as genetic makeup, sex, age and microbiological status – can impact the outcomes of preventive or curative probiotic treatment. We explore why and how these factors can influence the results of probiotic studies and negatively impact the reproducibility in animal experiments. These same factors might profoundly influence the outcomes of human clinical trials, and can potentially explain the conflicting results from probiotic intervention studies. Therefore, we also link these host-related factors to human probiotic study outcomes in the context of airway allergies.

## Abstract (precis)

**Summary:** This Review discusses the host factors that influence the outcomes of *in vivo* probiotic interventions in allergy, focusing on genetics, sex, age and microbiological status in mouse models of allergic disease.

## Introduction: linking microbiota, probiotics and allergic disease

Allergic diseases present a compelling challenge for public health owing to their increasing prevalence in both developed and developing countries. Approximately 1 billion people worldwide currently suffer from allergies, and these numbers are estimated to increase to 4 billion in the next 30-40 years (Akdis and Agache, 2014).

Allergy is defined as a hypersensitivity reaction caused by an immunological response to a specific antigen, referred to as an allergen (Ring, 2014). Widespread allergies – such as those to pollen, house dust mites, animal dander or certain foods – are generally immunoglobulin (Ig) E mediated (Akdis and Agache, 2014). These allergens initially interact with innate immune cells, and the subsequent immune cascade results in a T helper (Th; see Glossary, Box 1) 2 bias of the adaptive immunity, production of allergen-specific IgE and effector cell sensitization. Repeated contact with the allergen triggers mast cell and basophil activation and allergic mediator release, resulting in symptoms that vary from sneezing and itchy skin rashes to severe shortness of breath and anaphylaxis. Typical treatment options consist of either avoiding the causative allergen, or providing symptom relief with antihistamines and corticosteroids aimed at blocking the mediator activity and/or immune cell activation. Poorly managed allergic diseases are commonly associated with a high socioeconomic burden, owing to healthcare costs and productivity losses, as well as a diminished quality of life (Meadows et al., 2013).

**Box 1. Glossary**

**Adjuvant:** substance capable of boosting the immune response to an antigen.

**Airway hyperreactivity (AHR):** increased response of the airways to nonspecific stimuli, resulting in easily triggered airway constriction typical of asthma.

**Allergic sensitization:** process during which the immune system becomes hypersensitive to an allergen, linked to allergen-specific antibody production.

**Aluminium hydroxide (alum):** adjuvant often used in vaccines or to boost sensitization in animal models.

**Atopy:** predisposition of an individual to develop allergic disease.

**Bronchoalveolar lavage fluid (BALF):** saline solution collected after irrigation of the airways containing cells and fluid from the bronchi and alveoli.

**Colony-forming unit (CFU):** a commonly used measure of microbial load.

**Commensal:** organism exhibiting commensalism, a form of symbiosis between two species.

**Dendritic cell (DC):** immune cell capable of antigen presentation that forms a link between the innate and adaptive immunity.

**DNA fingerprint analysis:** analysis of variable DNA regions to determine the DNA profile unique to an individual.

**Enhanced pause (Penh):** dimensionless measurement reflecting airway obstruction through analysis of an airflow pattern in a live animal, e.g. during whole-body plethysmography.

**Fc receptor (FcR):** antibody receptor located on the surface of immune cells and capable of binding antibodies.

**Haematoxylin and Eosin (H&E) staining:** a common tissue staining method used to distinguish cellular structures.

**Innate lymphoid cell (ILC2):** innate immune cell that is lymphoid in morphology, but lacks antigen-specific receptors.

**Interleukin (IL):** signal protein belonging to the group of cytokines and involved in immune response regulation.

**Intestinal epithelial barrier:** property of intestinal mucosa responsible for containment of intraluminal molecules and regulation of their passage into the underlying lamina propria.

**Intragastrically (i.g.):** administered into the stomach.

**Intranasally (i.n.):** administered into the nostrils.

**Intraperitoneally (i.p.):** administered into the peritoneal or abdominal cavity.

**Jaccard similarity index:** statistical measure of similarity between two sample sets expressed as the intersection of the sets divided by their union.

**Mast cell:** white blood cell that contains granules with chemical mediators responsible for the typical symptoms of allergy.

**Major histocompatibility complex class II (MHCII):** molecular complex located on antigen-presenting cells that mediates antigen presentation and immune response initiation.

**Mesenteric lymph nodes (MLNs):** lymph nodes located in the mesentery (tissues that attach the intestines to the abdominal wall).

**Microbe-associated molecular patterns (MAMPs):** molecular structures of bacteria recognized by receptors on host cells, resulting in immune effects on the host.

**Ovalbumin (OVA):** abundant egg white protein, one of the major egg allergens.

**Ovariectomized:** a term used to describe a woman or a female animal whose ovaries have been surgically removed.

**Pattern recognition receptors (PRRs):** receptors on innate immune cells of the host that recognize microbe-associated molecular patterns.

**Periodic acid–Schiff (PAS) staining:** tissue staining method for detection of polysaccharides and mucosubstances.

**Peyer's patches:** isolated lymphoid follicles found in the small intestine.

**Prebiotic:** food ingredient promoting development and activity of beneficial organisms within the host.

**Regulatory T cell (Treg):** a type of white blood cell capable of regulating or suppressing immune responses and promoting immunological tolerance.

**Specific-pathogen-free (SPF):** (laboratory animals) free of certain microorganisms and parasites belonging to a defined list.

**Subcutaneously (s.c.):** administered under the cutaneous skin layer.

**Steroids (inhaled):** medication used to treat inflammation, such as asthmatic inflammation of the airways.

**T helper (Th) cell:** a type of white blood cell capable of recognizing antigens presented by antigen-presenting cells and releasing cytokines that regulate the immune response.

**Tolerogenic:** leading to induction of immunological tolerance.

**Toll-like receptor (TLR):** a type of PRR characterized by the presence of a toll-IL-1 receptor (TIR) domain.

**Tumor necrosis factor (TNF):** a cytokine involved in inflammation signalling.

**Whole-body plethysmography:** technique used for measuring respiratory function in live subjects placed in a sealed chamber by recording pressure changes within the chamber.

The currently rising incidence of allergic disease has tentatively been explained by the ‘hygiene hypothesis’, which links a general decrease in microbial exposure of children in developed countries to reduced immune system stimulation and subsequent Th1/Th2 imbalance, which predisposes to allergic diseases (Strachan, 2000). Mammals house a numerous and diverse community of microorganisms – including bacteria, fungi, archaea, protists and viruses – collectively entitled the microbiota, that is constantly involved in a dynamic molecular and cellular crosstalk with the host (Rooks and Garrett, 2016). The microbiota plays a crucial role in the balanced development of the human immune system throughout our lives, and especially during the establishment of immune functions in early childhood (Guarner et al., 2006; Rooks and Garrett, 2016; Torow and Hornef, 2017). A more recent ‘old friends’ hygiene hypothesis states that specific types of organisms are required for the proper development of the human immune system, such as helminths, but also microorganisms, including bifidobacteria, lactobacilli and saprophytic mycobacteria (Rook et al., 2003).

These organisms are thought to have been part of our microbiota during human evolution and to have co-evolved with the human immune system to promote immune tolerance. This includes regulatory T cell (Treg) (Box 1) induction, and the control of the Th2 and Th1 balance, thus contributing to the prevention of both allergic and autoimmune disease development (Lee and Mazmanian, 2010; Umetsu et al., 2002). The crucial role of the interactions between the microbiota and the human immune system prompted attempts to positively steer and balance the development of immune functions by administering specific beneficial live microorganisms, or probiotics. Probiotics are ‘live microorganisms that, when administered in adequate amounts, confer a health benefit on the host’ (Hill et al., 2014). Various clinical studies examined the potential influence of probiotic strains in the context of allergic disease, but, so far, the results have been conflicting and few practical recommendations could be formulated regarding probiotic interventions in allergy (Forsberg et al., 2016). Prevention is especially important in allergic airway disease, as the underlying driving factors are often established in childhood, while the development of symptoms might take years or even decades (Umetsu et al., 2002). Recent meta-analyses of probiotic intervention studies point to moderate beneficial effects for primary eczema prevention (Cuello-Garcia et al., 2015; Zuccotti et al., 2015), especially when probiotics are administered both before and after birth to the mother and the infant. This type of perinatal intervention was also successful in reducing the incidence of allergic sensitization (Box 1), which was not the case for pre- or postnatal treatment alone (Zhang et al., 2016). However, the evidence for probiotic prevention of allergic airway disease remains scarce, as no significant effect on wheezing episodes or asthma development could be demonstrated in children (Azad et al., 2013).

The conflicting results might arise from differences in study design, read-outs and patient stratification. One major limitation for a comprehensive meta-analysis of probiotic studies is the implementation of different probiotic species and strains, most commonly *Lactobacillus* or *Bifidobacterium*, or mixes thereof (Zuccotti et al., 2015). The administered probiotic doses also vary greatly between the studies, ranging from 10^7^ to 10^10^ or more colony-forming units (CFU)/day, and the treatment might last from several months to several years (Zuccotti et al., 2015). However, even when using the same probiotic strain and a similar administration regimen, the results can differ between clinical studies, underlying the potential importance of host-related factors. For instance, maternal and/or perinatal administration of *Lactobacillus rhamnosus* GG reduced childhood eczema in several studies (Kalliomäki et al., 2001, 2007), but this effect was not always significant in other study populations or when using other probiotic formulations (Kopp et al., 2008; Ou et al., 2012). Therefore, clinical study heterogeneity remains a major barrier for the formulation of evidence-based guidelines on probiotic implementation in allergies (Forsberg et al., 2016).

In this Review, we discuss the host factors that might be responsible for the heterologous outcomes observed in studies involving probiotic administration in the context of allergies. The focus lies on models of respiratory allergies, for which probiotic interventions remain especially controversial. Furthermore, airway allergy prevention and treatment exemplify the complexity of probiotic interactions with various host organ systems, including the respiratory system and the host immunity. We will thus discuss how these interactions can be influenced by host genetics, sex, age and resident microbiota.

## The use of mouse models in probiotic research

Because the immune system's intrinsic complexity cannot yet be fully reproduced *in vitro*, animal models are an indispensable tool to investigate the effects and especially the mechanisms of action of various probiotic strains on the immune system (Martín et al., 2017). Certain manipulations not available in humans, such as invasive procedures, targeted disruption of immune function, use of transgenic host strains and the possibility to test genetically modified probiotics, are just a few advantages that animal models can offer (Herz et al., 2004). Furthermore, the current regulatory framework might prevent studies of novel probiotic strains in humans prior to a thorough assessment of their safety and therapeutic risks, especially when they involve genetically modified bacteria. Among various model organisms, mice are preferred owing to their small size and subsequent ease of handling and housing, short generation time, and the availability of immunological tools and relevant transgenic mouse strains (Sagar et al., 2015; Shay et al., 2013). Mice also show a relatively high anatomical and physiological similarity to humans, which makes it possible to use them for investigating the effects of probiotics on immune parameters involved in the prevention and treatment of allergic diseases (Shay et al., 2013). Studies in mice can be used for initial testing of safety and potential beneficial effects of probiotics, as well as to clarify, support or further investigate the mechanistic hypotheses based on findings from microbiota-related studies in humans. Such animal studies can provide crucial insight into the microbe-host interactions that could not be obtained in clinical trials (Martín et al., 2017). Studies on the associations between probiotics, the microbiome and immune-related studies can include other mammalian models, such as guinea pigs (Tsunemine et al., 2015), pigs (Thomas et al., 2011), dogs (Marsella et al., 2013) and macaques (Hirao et al., 2014). However, these nonmurine models are beyond the scope of this Review.

### Mouse models of allergic airway disease

The pathophysiology of allergic disease results from a complex sequence of events involving various innate and adaptive immune mechanisms that lead to allergic sensitization and subsequent allergic inflammation upon re-exposure to allergens. A wide variety of mouse models have been developed for studying allergies, most of which use ovalbumin (OVA; Box 1) for allergic sensitization (Hellings et al., 2006; Huvenne et al., 2010; Nials and Uddin, 2008). However, as OVA is predominantly a food allergen, it might not be especially suitable for modelling other types of allergy, such as allergic airways disease. In that case, models implementing clinically relevant aeroallergens, such as house dust mite proteins (Steelant et al., 2016) and tree pollen (Schabussova et al., 2012; Shimada et al., 2005), might have a higher translational relevance.

**Fig. 1.**
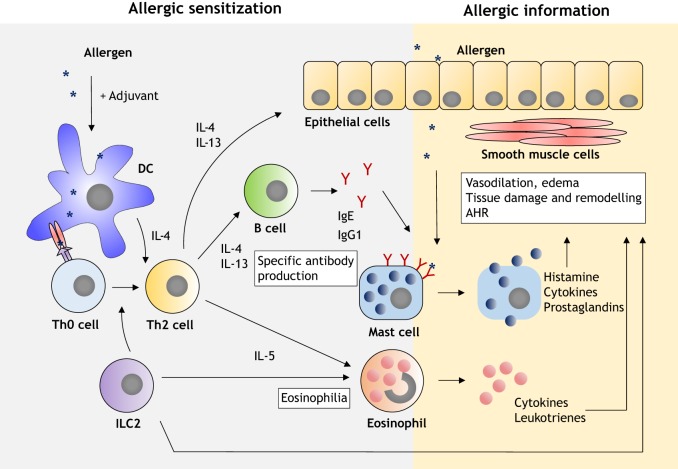
**Mechanisms of allergy induction in murine models.** During allergic sensitization, antigen-presenting cells (e.g. DCs) induce the generation of Th2 cells (Humeniuk et al., 2017). Th2 cytokines IL-4, IL-5 and IL-13 promote eosinophil recruitment (eosinophilia) and induce tissue inflammation, which is exacerbated by type 2 innate lymphoid cells (ILC2; Box 1) action. Th2 cells also interact with B cells to induce the production of allergen-specific IgE and IgG1, which bind to effector cells (e.g. mast cells) at mucosal surfaces (Galli and Tsai, 2012; Nials and Uddin, 2008). Allergic inflammation is induced upon repeated contact with the allergen, which promotes the release of inflammatory mediators affecting the surrounding tissues and leading to symptom development (Nials and Uddin, 2008).

During allergic sensitization, allergens can be administered via various routes; for example, intraperitoneally (Karimi et al., 2009; Mendes et al., 2017; Pellaton et al., 2012; Yu et al., 2010), epicutaneously (Kim et al., 2014) and subcutaneously (Schwarzer et al., 2013; Jan et al., 2012), or intratracheally (Shalaby et al., 2013). This is followed by uptake and processing of the allergen by antigen-presenting cells, such as dendritic cells (DCs; Box 1, Fig. 1) (Humeniuk et al., 2017). The resulting peptides are presented in the context of major histocompatibility complex class II (MHCII; Box 1) to naïve T helper (Th0) cells. This leads to their priming and differentiation into Th2 cells and production of Th2 cytokines, such as interleukin (IL; Box 1) 4, IL-5 and IL-13, which further drive allergic sensitization and tissue inflammation (Nials and Uddin, 2008). Contact with allergens also sensitizes B cells. The Th2 cytokines IL-4 and IL-13, as well as the direct interaction of Th2 cells and of follicular T helper cells with B cells located in the lymphoid tissues or at mucosal surfaces, leads to B cell class-switch recombination and production of allergen-specific IgE and IgG1 (Galli and Tsai, 2012; Nials and Uddin, 2008). In mice, IgG1 is typically induced in Th2 responses and is highly important in the allergic reactions (Nials and Uddin, 2008). Allergen-specific antibodies subsequently bind to high-affinity Fc receptors (FcRs) (Box 1) on the surface of effector cells in the airway mucosa, such as mast cells (Box 1), thus sensitizing them for mediator release upon contact with the allergen (Galli and Tsai, 2012).

As mice do not spontaneously develop serious allergic disease, stronger stimulation of the immune system is often required to obtain a stable representation of the human condition (Nials and Uddin, 2008). To additionally stimulate the Th2 response and to develop a more pronounced allergic sensitization, many models require the application of effective adjuvants (Box 1). Aluminium hydroxide (alum; Box 1) is commonly used for intraperitoneal sensitization (Karimi et al., 2009; Kozakova et al., 2016; Pellaton et al., 2012), staphylococcal enterotoxin B (SEB) for intranasal allergen exposure (Hellings et al., 2006; Huvenne et al., 2010), or cholera toxin (CTX) for oral sensitization (Liu et al., 2017). However, this strong artificial stimulation of the immune system can potentially skew the results of the probiotic treatment and prevent their correct extrapolation to clinical studies for two reasons. First, the forced sensitization will be less susceptible to manipulation by probiotics. Second, humans are sensitized by airway or oral exposure to natural allergens, often without strong adjuvants. This restriction certainly applies to sensitization through intraperitoneal or subcutaneous injections, which might lead to strong antibody production in mouse models, but with the involvement of immune mechanisms not typically implicated in airway allergy development in humans (Kool et al., 2008). This can impair the results of probiotic studies in such models, as the probiotic treatment effects are often subtle. It might, therefore, be more desirable to sensitize mice with natural whole allergen extracts, such as house dust mite extracts (Steelant et al., 2016), which have intrinsic adjuvant properties.

After sensitization, a challenge step induces allergic inflammation, which involves re-exposure of the animal to the allergen via the administration route typical for a given allergic disease. For instance, in allergic airway disease models, an inflammatory response is evoked in the airways by repeated exposures (or challenges) to the allergen, through either aerosol inhalation (Mendes et al., 2017; Pellaton et al., 2012) or intranasally (Nunes et al., 2018; Wu et al., 2016). We list examples of such models in Table 1. Upon allergen re-exposure, effector cells release pro-inflammatory cytokines and proteases, which attract eosinophils and neutrophils, induce an inflammatory process, weaken the epithelial barrier and lead to tissue damage, especially upon repetitive exposure (Galli and Tsai, 2012; Nials and Uddin, 2008). This release of pro-inflammatory mediators in the airways also leads to airway hyperreactivity (AHR; Box 1), hypersecretion of mucus, airway tissue remodelling and other manifestations reminiscent of asthma-associated chronic allergic airway inflammation in humans (Nials and Uddin, 2008). As a result, allergic airway inflammation is typically assessed based on differential counts of inflammatory cells, such as eosinophils and neutrophils, in the bronchoalveolar lavage fluid (BALF; Box 1) (Jan et al., 2012; Karimi et al., 2009; Nunes et al., 2018; Pellaton et al., 2012) (Table 1). Airway function is assessed based on airway resistance measurements with invasive (MacSharry et al., 2012) and noninvasive (e.g. whole-body plethysmography; Box 1) (Jan et al., 2012; Karimi et al., 2009; Wu et al., 2016) techniques to assess changes in AHR. In addition, histopathological evaluation of the airways is typically performed, with Haematoxylin and Eosin staining to examine the degree of inflammatory cell infiltration and periodic acid–Schiff (PAS) staining (Box 1) to assess mucus production (Karimi et al., 2009; Nunes et al., 2018).

**Table 1.**
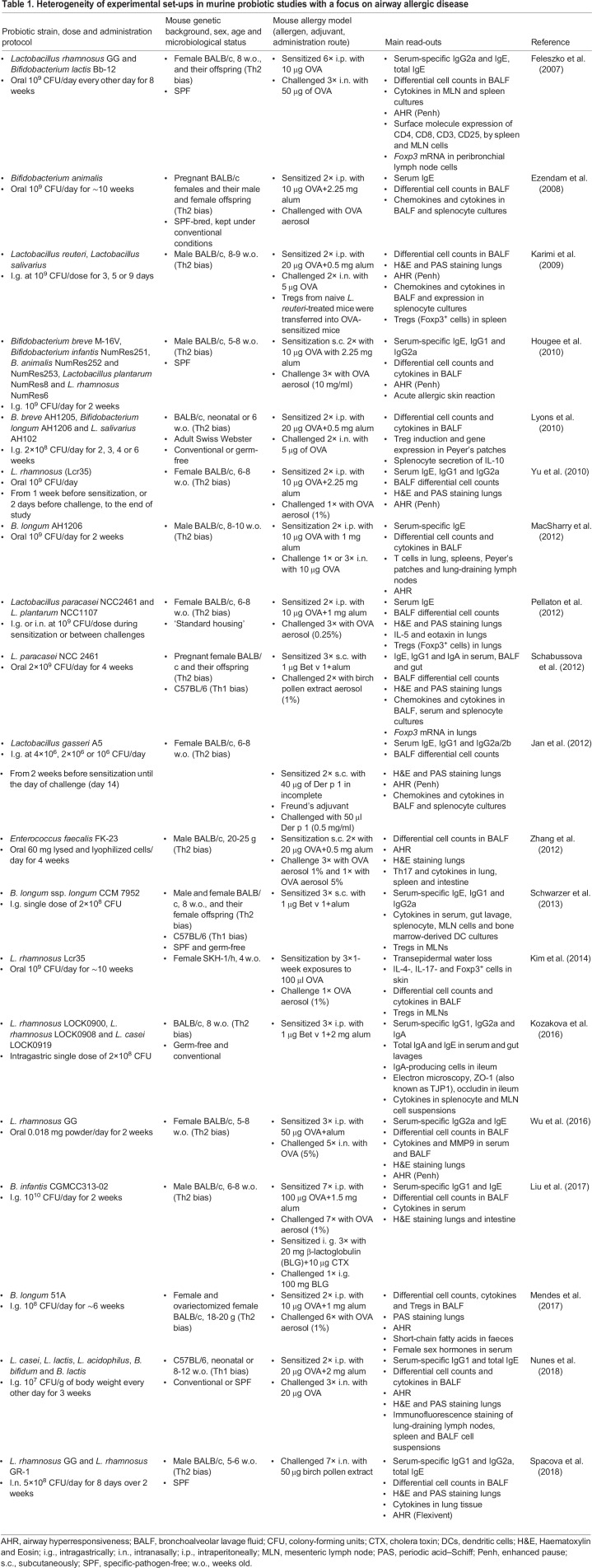
**Heterogeneity of experimental set-ups in murine probiotic studies with a focus on airway allergic disease**

### Probiotic administration in mouse models of allergic disease

Administration of probiotics in mice has been explored for preventing or reducing the development of the hallmarks of allergic disease in animal studies (Table 1). To gain insight into the probiotic- and allergy-associated mechanisms responsible for the observed effects, researchers analysed several parameters, such as allergen-specific antibody production (Pellaton et al., 2012), Th1, Th2 and Treg cytokine levels in the airways and lymph nodes (Jan et al., 2012; Karimi et al., 2009; Pellaton et al., 2012), effector and regulatory T cell population counts (Karimi et al., 2009; Pellaton et al., 2012), and airway function (Mendes et al., 2017; Nunes et al., 2018; Wu et al., 2016). Similar to human clinical trials (Forsberg et al., 2016), protocols for probiotic mouse studies for prevention and treatment of allergic disease are substantially heterogeneous regarding the route, dose, timing, probiotic strain or strain mixture administration (Table 1). The timing of probiotic administration can vary greatly, both relative to the induction of allergic disease, as well as to the duration of probiotic intervention. Probiotic strains have been administered before, after, or during allergic sensitization or challenge. Preventive administration before sensitization remains the most common method used (Kozakova et al., 2016; Wu et al., 2016; Yu et al., 2010) (Table 1), similarly to the set-ups of human clinical trials (Boyle et al., 2009; Forsberg et al., 2016). However, in some mouse studies the treatment is continued throughout allergy induction (Kim et al., 2014; Pellaton et al., 2012; Wu et al., 2016) (Table 1). The duration of probiotic treatment can be as short as a few days (Karimi et al., 2009), but most often lasts for several weeks (Pellaton et al., 2012), with some studies continuing the administration throughout the whole life of the animal (Feleszko et al., 2007). As in human trials, the probiotic administration route is typically oral (Jan et al., 2012), with doses ranging from 10^6^ (Jan et al., 2012) to 10^9^ (Karimi et al., 2009; Pellaton et al., 2012) CFU or 2×10^9^ CFU per mouse (Schabussova et al., 2012) (Table 1). The dose can, however, go up to 10^10^ (Liu et al., 2017) or even 10^11^ CFU per mouse, such as when studying probiotic effects on antibody levels in the context of vaccination (Esvaran and Conway, 2016). Probiotics are usually administered orally via drinking water or food (Lee et al., 2016; Schabussova et al., 2012), or through intragastric intubation when more precise dose control is preferred (Karimi et al., 2009; Pellaton et al., 2012). Researchers are increasingly exploring intranasal administration of probiotic bacteria in both mouse and human studies, as it often proves more effective in modulating allergic airway inflammation (Spacova et al., 2018; Pellaton et al., 2012). In addition to probiotic-related factors, experimental set-ups of probiotic studies substantially differ in mouse-related parameters. This is the case even in the narrower context of a single disease, such as allergic airway inflammation, as demonstrated in Table 1.

## Critical host-related factors

**Fig. 2.**
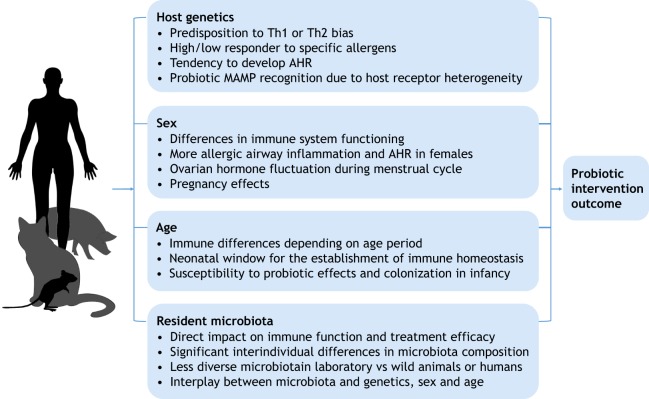
**Host factors that can influence the outcome of probiotic intervention studies in the context of allergic disease.** The influence of each factor is described in more detail in the corresponding sections of this Review. AHR, airway hyperreactivity; MAMP, microbe-associated molecular pattern.

Host-related factors can have a profound influence on the functioning of the immune system in the context of allergic disease and microbe-host interactions (Laukens et al., 2016). Several potentially critical parameters, such as host genetic makeup, age, sex and microbiological status, can vary within both animal and human experimental set-ups (Laukens et al., 2016; Martín et al., 2017) (Fig. 2). Awareness of factors other than the probiotic strain and its application protocol is crucial for correct animal study design. Indeed, these host-related factors can have a profound influence on the functioning of the immune system in allergic airway disease and microbe-host interactions. The availability of studies focusing specifically on the influence of genetic makeup, age, sex and microbiological status of the host on the study outcome is currently limited. Here, we provide evidence of how these factors influence the functioning of the immune system and potentially also the effect of probiotics.

### Host genetics

Inbred laboratory mouse strains are routinely used to explore probiotic modulation of host immune responses in the context of airway allergy. Inbred strains offer genetic homogeneity and subsequently limited variation within the strain. However, notable differences in immune system functioning between various inbred mouse strains have been described (Ewart et al., 2000; Sellers et al., 2012; Wells et al., 2003). The resulting divergent immune response phenotypes can profoundly influence probiotic study outcomes. Differences in genetic background can potentially also result in distinct signalling patterns in probiotic-host cell interactions.

Inbred mouse strains can be classified into strong and intermediate/low responders based on their tendency to develop allergen-specific IgE/IgG1 antibodies and allergic airway inflammation, which is often linked to a strain-specific Th1 or Th2 bias (De Vooght et al., 2010; Sellers et al., 2012). BALB/c, A/J and BP2 mice lean predominantly towards Th2-type immune reactions upon sensitization and challenge, whereas C57BL/6, AKR, CBA and B10D2 mice are more prone to mounting Th1-type responses (Table 1) (De Vooght et al., 2010; Herz et al., 2004). Consequently, BALB/c mice are high Th2 responders to a wide range of allergens, including the model allergen OVA and birch pollen extract, while strains such as C57BL/6 and CBA tend to develop low to intermediate allergen-specific Th2 responses to these allergens. Despite this, C57BL/6 are high responders to house dust mite extract and ragweed (Herz et al., 2004). Immunological differences also inform the level of inflammatory responses in the lung, with BALB/c mice having more prominent inflammatory cell influx and elevation of IL-4, IL-5 and tumor necrosis factor (TNF; Box 1) in the airways compared with C57BL/6J mice (Herz et al., 1998). Likewise, significant lung eosinophilia was observed in A/J and C3H/HeJ mice upon OVA sensitization and challenge compared with AKR/J and C57BL/6J mice (Ewart et al., 2000). Furthermore, inbred mouse strains diverge in their ability to develop AHR (De Vooght et al., 2010; Ewart et al., 2000; Herz et al., 1998; Shinagawa and Kojima, 2003), which is another hallmark of allergic asthma. Sensitized BALB/cJ, A/J and AKR/J mice showed a significant increase in AHR upon OVA airway challenge, whereas the changes in AHR were much less pronounced or virtually absent in C57BL/6J and C3H/HeJ mice (Ewart et al., 2000; Herz et al., 1998).

Mouse strains also differ in their interactions with intestinal microbiota and responses to microbe-associated molecular patterns (MAMPs; Box 1) involved in bacteria-host immune cell interactions. Differences in interaction with noninvasive bacteria between the BALB/c and C57BL/6 strains have been linked to elevated production of polyreactive IgA antibodies and B1a cells in the spleen and Peyer's patches (Box 1) of BALB/c mice (Fransen et al., 2015). Likewise, distinct variations in gene expression patterns were observed between macrophages from C57Bl/6J, DBA2, BALB/c, C3H/ARClpsn and C3H/HeJlpsd mice upon lipopolysaccharide challenge (Wells et al., 2003). Numerous genetic loci that can affect microbe-host interactions were involved, including those responsible for Toll-like receptor (TLR; Box 1) 4 and arginase production. In fact, the genetic background of the mouse can determine its gut microbiota composition, possibly due to mouse strain-specific host-microbe interactions which favour the presence of certain microorganisms. DNA fingerprint analysis (Box 1) of gut microbiota in eight recombinant inbred mouse lines derived from the Collaborative Cross project has shown that the genetic background of the mouse was a more prominent determinant of gut microbial composition than the sex or maternal influences (Kovacs et al., 2011). In this study, the Jaccard similarity index (Box 1) showed higher microbiota composition similarity values between faecal microbiota DNA pools of the same inbred line than for those of the same sex. Another study found that genetic variation between mouse strains was responsible for ∼19% of the variance in their intestinal microbiota (Hildebrand et al., 2013). It is therefore possible that not only individual differences between mice, but also their genetic make-up, play a determining role in host-microbe interactions.

Consequently, mouse strain-specific differences in immune responses and microbe-host interactions can indeed influence probiotic intervention studies. Notable gene expression differences in the small intestine and colon between C57BL/6 and BALB/c mice were observed after a 4-week administration of the VSL#3 probiotic mixture containing eight bacterial strains of *Lactobacillus*, *Bifidobacterium* and *Streptococcus* (Mariman et al., 2015). These differences were linked to the presence or absence of a Th2 and Th17 bias in the mouse strains. For example, BALB/c mice showed increased expression of Th2-linked transcription factor GATA3 in the small intestine, in contrast to C57BL/6 mice, which had higher transcription levels of RORγt (Rorc) associated with Th17 immune responses (Mariman et al., 2015). In another study, a combination of the probiotic *Bifidobacterium longum* and prebiotic (Box 1) pectin fibre was able to reduce airway inflammation and AHR in A/J mice, but no similar effects were observed in C57BL/6 mice (Ferreira et al., 2016). The authors linked these differences to lower microbiota diversity in A/J mice, and suggested that the intrinsic microbiota of the host can interfere with the expected probiotic effects. Likewise, local peritoneal accumulation of eosinophils induced by the administration of a Japanese cedar pollen solution was reduced following treatment with the lysed probiotic *Enterococcus faecalis* FK-23 in BALB/c, C3H/HeN and C3H/HeJ mice, but not in C57BL/6 mice, possibly due to the differences in TLR functionality between the strains (Shimada et al., 2005). However, certain mouse strain-specific effects can be compensated by adjusting the treatment parameters, such as the probiotic dose. Although the effect of *Lactobacillus fermentum* PC1 as a mucosal adjuvant differed between BALB/c and DBA/1 mice at lower doses (10^8^ CFU), higher doses (10^11^ CFU) led to a robust Th1 response regardless of the host genetic background (Esvaran and Conway, 2016).

Hence, the genetically determined immune response patterns must be taken into account when investigating the effects of probiotic interventions, and the wide range of inbred mouse strains available can serve as a useful tool to understand the variations in immune response present in the human population.

### Sex

Epidemiological and clinical studies suggest that sex influences the incidence and pathology of respiratory allergic diseases, including allergic asthma (Choi, 2011; Leynaert et al., 2012). Consistently, a number of animal studies point to immunological differences in the development of respiratory allergies between male and female mice. Female mice generally show higher IgE serum levels than males (Bonnegarde-Bernard et al., 2014; Corteling and Trifilieff, 2004; Melgert et al., 2010). Several studies also point to a more pronounced airway inflammation and AHR (Bonnegarde-Bernard et al., 2014; Melgert et al., 2010) after sensitization and challenge with model allergens such as OVA and house dust mite extract in females. This sex disparity has been linked to ovarian hormones, such as progesterone, increasing the severity of allergy-associated reactions, including airway eosinophilia and AHR (Hellings et al., 2003). In contrast, ovariectomized (Box 1) animals sensitized and challenged with OVA showed decreased production of IL-4, IL-5, IL-13 and IL-17, and an increase in IL-10 in the lung (Ligeiro de Oliveira et al., 2013). However, the time of ovariectomy relative to allergen administration might be crucial, as another study demonstrated that ovariectomy after initial allergen exposure led to an increase in allergic airway inflammation and a decrease in airway function upon re-challenge of mice with the same allergen (Mendes et al., 2017). Consequently, it is plausible that due to these differences, sex can have a profound influence on the outcomes of probiotic treatment. Sex indeed affects the efficacy of anti-inflammatory agents such as inhaled steroids (Box 1) in allergic asthma, both in mice (Corteling and Trifilieff, 2004) and in humans (Choi, 2011).

Although a significant number of probiotic studies involving mouse models of allergic inflammation favours the use of female mice (Table 1), the potential influences of sex hormones on immunomodulation by probiotics remain elusive. Differences in sex hormones influence parameters associated with beneficial probiotic action in airway allergies, such as Treg induction (Feleszko et al., 2007; Karimi et al., 2009). However, elucidating the effects of female hormones on Treg levels remains challenging owing to the hormonal changes associated with the menstrual cycle and pregnancy in both humans and mice. Studies in mice showed that naïve nonimmunized ovariectomized females display elevated frequencies of lymph node and spleen CD4^+^Foxp3^+^ Tregs (Ligeiro de Oliveira et al., 2013), while another study demonstrated similar Treg levels and function in the airways of male and female mice challenged with OVA (Melgert et al., 2010). OVA challenge led to higher numbers of effector T cells in the lungs of females, possibly resulting from a greater abundance of alternatively activated macrophages (Melgert et al., 2010). Other innate and adaptive immunological mechanisms potentially involved in probiotic action have also been shown to be sex dependent (Klein and Flanagan, 2016). For example, male murine macrophages display higher surface levels of CD14 (Marriott et al., 2006), a co-receptor involved in TLR-mediated probiotic-host interactions (Lebeer et al., 2010).

The influence of sex hormones on microbe-host interactions is further supported by notable differences reported in gut microbiota between adult male and female mice, which become less significant after male castration (Yurkovetskiy et al., 2013). Sex effects have also been observed specifically in the context of probiotic interventions in mouse models. For example, pretreatment with *Bifidobacterium animalis* before the induction of OVA-mediated airway allergic inflammation led to a more pronounced lowering of Th2 cytokine levels in stimulated spleen cells of male mice compared with females (Ezendam et al., 2008). *In vivo*, this could lead to a less Th2-skewed environment in lymphoid tissues, and thus diminished allergy symptom development (Fig. 1). This sex difference is potentially linked to the fact that spleen cells isolated from OVA-sensitized females elicit a stronger Th2 cytokine response upon *in vitro* stimulation, which might mask the subtle immunomodulatory effects of *B. animalis* pretreatment. Ovarian hormone levels fluctuate dramatically during the murine oestrous cycle, which can additionally skew individual responses to airway allergy induction and treatment. In another study, treatment with *Lactobacillus reuteri* BM36301 led to a significant reduction in pro-inflammatory TNF in the sera of aged female mice, whereas this was less pronounced in the treated males (Lee et al., 2016). However, when testing the effects of another potential probiotic, *L. reuteri* 6475, on intestinal inflammation and bone formation, jejunal and ileal TNF mRNA levels and bone density were markedly improved only in the male group (McCabe et al., 2013). The differences in outcomes between the two studies might be attributed to the differences in experimental set-ups, such as the choice of probiotic strains, dosage and administration frequency, as well as mouse age at the beginning and end of the experiment.

### Age

Age-dependent qualitative and quantitative differences in the innate and adaptive immune responses have long been recognized in vertebrates, with the pre- and early postnatal period representing a key point in the development of the mammalian immune system (Torow and Hornef, 2017). This critical window for the establishment of immune homeostasis has been observed in both humans and animal models, defining a time in early life during which the immune system is particularly susceptible to the influence of exogenous factors, including those linked to microbiota alterations (Arrieta et al., 2015). In particular, microbial colonization of germ-free mice during the period up to 1 week postweaning could fully protect them from a hyper-IgE response later in life, but this effect was absent in mice that were colonized at a later age (Cahenzli et al., 2013). Similarly, exposure to the antibiotic vancomycin leads to diminished microbial diversity and subsequent exacerbation of allergic asthma in neonatal mice, whereas this is not the case for adult animals (Russell et al., 2012). Studies in mice have also demonstrated that immune functions potentially involved in microbe-host interactions, such as the innate immune recognition of bacterial components, are age dependent and active even before birth. For example, in mice, intestinal expression patterns of TLR change during the late gestation period, as TLR4 expression increases and TLR9 expression decreases (Gribar et al., 2009). This is followed by an ageing-related drop in TLR function that could be explained by reduced functional receptor levels on macrophages (Renshaw et al., 2002). Notably, significant changes in TLR4 and TLR9 expression can occur in a matter of days during the early stages of murine immune system development (Gribar et al., 2009), which underlines the importance of age standardization in mouse experiments. In addition, the adaptive mucosal immune system shows marked differences in cell composition and function between neonatal and adult mice (Torow et al., 2015). For instance, neonatal CD4^+^ T cells in the small intestine maintain an immature phenotype until weaning, with Tregs and maternal IgA playing an important role at this stage.

Results from mouse studies demonstrate that beneficial effects of bacterial strains, such as the induction of Treg expansion by *Bifidobacterium breve* AH1205, are only possible when the bacteria are administered in infancy (Lyons et al., 2010). This suggests that not all beneficial effects of probiotic interventions observed in children can be extrapolated to the adult population. However, this largely depended on the strain used, as *B. longum* AH1206 increased Treg levels in both infant and adult mice, whereas *L. salivarius* AH102 administration affected neither (Lyons et al., 2010). Interestingly, *B. longum* AH1206 conferred protection against OVA-induced Th2 sensitization and airway inflammation, while the other strains did not. The probiotic strain *L. paracasei* NCC 2461 also modulated allergic airway inflammation in mice, even when administered as early as the perinatal and lactation period, pointing to the importance of early microbial exposure in the development of the immune system of the pups (Schabussova et al., 2012). These effects were linked to stimulation and transfer of immunoregulatory mechanisms in the mother and the offspring, as the Th1/Th2 balance during the fetal and neonatal stages can influence the development of the immune system later in life.

In some cases, the differences in immunomodulatory effects of administered probiotics between infant and adult mice can potentially be attributed to the less efficient colonization capacities of certain probiotic strains in adults. For example, *Lactobacillus johnsonii* Ms1 remained in the murine gastrointestinal tract for at least 7 days after the last administration in neonatally exposed mice, but not when they received the bacteria at 2 or 7 weeks of age (Ozawa et al., 2012). In the same study, neonatal or perinatal administration of *Lactobacillus plantarum* No. 14 and *L. plantarum* JCM did not result in gut colonization of the pups, although high levels of these bacteria could be detected in the faeces of the corresponding females (Ozawa et al., 2012). Taken together, these results demonstrate that certain probiotic effects and their magnitude can be influenced by age-specific functions, such as immunological and gut barrier maturity of the host (Lyons et al., 2010), which should be taken into account when determining the window of probiotic intervention.

### Resident microbiota

Once probiotics are administered to the host, they interact with the resident microbiota, and can work in conjunction or compete, depending on the characteristics of the strain and the properties of the established microbial community. The importance of the resident microbiota in human and animal studies is becoming increasingly recognized as one of the factors that can influence study outcomes and experimental reproducibility (Laukens et al., 2016). Large-scale faecal and airway microbiota assessment is thus becoming common in the context of experimental allergic disease (Remot et al., 2017) and other immunological research in murine models (Beura et al., 2016; Hildebrand et al., 2013). As a result, there is increasing evidence that the steady-state microbiota composition of animals housed under the same experimental conditions is not necessarily similar. In fact, interindividual variation between mice is the most important determinant of differences in murine gut microbial communities (Hildebrand et al., 2013). In addition, housing mice in the same cage synchronized their gut microbiota composition (Hildebrand et al., 2013). Similar results were obtained in a recent study, where inbred 129X1/SvJ mice housed under the same controlled conditions demonstrated significant variation in microbiota composition explained by the effects of individual mice, shipment group and co-housing (Hoy et al., 2015). These differences in resident microbiota can profoundly impact experimental outcomes in allergic disease research, especially because different commensal (Box 1) members of the murine microbiota are capable of either promoting or preventing allergic disease (Remot et al., 2017). In addition, certain members of the human microbiota, such as the gut isolate *L. rhamnosus* Lcr35 (Jang et al., 2012), various *Bifidobacterium* isolates (López et al., 2011) and other healthy human faecal isolates (Faith et al., 2014) have been shown to induce tolerogenic (Box 1) immune responses when transplanted in experimental mouse models and in *in vitro* studies. The magnitude of the probiotic effect might therefore depend on the interplay between probiotic- and resident microbiota-derived signals influencing the immune system in the context of allergy development.

Many probiotic studies in allergy models are conducted in mice that are specific-pathogen-free (SPF; Box 1) (Feleszko et al., 2007; Hougee et al., 2010; Schwarzer et al., 2013) or even germ-free (Kozakova et al., 2016; Lyons et al., 2010; Schwarzer et al., 2013). However, natural microbial complexity might be crucial in generating an appropriate mouse model for allergic disease, as such complexity is also intrinsic to humans. It has previously been observed that laboratory mice have a 1.3- to 1.5-fold reduction in the number of intestinal microbial genera compared with wild animals, which might have consequences for their immune status (Linnenbrink et al., 2013). Indeed, C57BL/6 inbred mice housed in hygienic SPF barrier facilities have an underdeveloped immune system, characterized by a scarcity of differentiated memory CD8^+^ T cell subsets, which is more typical of newborn than adult humans (Beura et al., 2016). Co-housing of these mice with non-SPF pet store animals led to an increase in effector T cell differentiation and distribution, making their immune system more similar to that of adult humans and outbred mice. Recent developments in microbiota research offer additional possibilities for obtaining mouse models that more closely resemble human adults; for example, through the use of human microbiota-associated mice, which received faecal transplantation from human subjects (Arrieta et al., 2016). Recapitulating the adult human immune traits in mice through alterations of their resident microbiota might, therefore, be crucial for increasing the predictive value of mouse probiotic studies in future clinical trials in the adult human population.

Germ-free mice demonstrate even more pronounced imbalances in the development of their immune system, such as a significantly increased tendency towards Th2 cytokine and antibody production, and decreased regulatory responses (Kozakova et al., 2016; Rodriguez et al., 2011), as well as an underdeveloped intestinal epithelial barrier (Kozakova et al., 2016). Signals from commensal bacteria can restore the immune system balance, for example, by modulating the circulation of inflammatory cells through IgE-mediated mechanisms, and profoundly influence allergy-related hematopoietic functions in the bone marrow in mice (Hill et al., 2012). This can be achieved not only by the addition of commensal microbial communities, but also by colonization with one or several probiotic strains. For instance, neonatal monocolonization of mice with *B. longum* ssp. *longum* CCM 7952 before subsequent allergic sensitization led to attenuation of IgE levels and Th1 and Th2 cytokine production by restimulated splenocytes, as well as an increase in serum regulatory cytokine levels (Schwarzer et al., 2013). Similarly, colonization with a mixture of *L. rhamnosus* LOCK0900, *L. rhamnosus* LOCK0908 and *L. casei* LOCK0919 restored the intestinal epithelial barrier (Box 1) and decreased allergen-specific serum IgE and IgG1 production, which was likewise linked to increased regulatory cytokine levels in serum and restimulated splenocytes (Kozakova et al., 2016). Interestingly, the influence of the microbiota is not uniformly significant in all genetic backgrounds. Although microbial colonization could inhibit high IgE production associated with the germ-free status in the inbred BALB/c and C57BL/6 strains, the absence of microbial colonization did not increase IgE levels in the outbred Swiss Webster and NMRI mice, possibly due to immunocompensatory mechanisms (Cahenzli et al., 2013). Nevertheless, insights into the interplay between resident microbiota composition and the administered probiotics in the context of airway disease are currently limited, and no doubt deserve further investigation.

## Link with human clinical studies and future prospects

Animal models can be used for the exploration of the effects of probiotics and their mechanisms of action, which is currently not possible in humans owing to unknown risks and ethical concerns. However, the ultimate goal is to provide useful insights that can be applied to the prevention and treatment of allergic disease in humans. A number of parallels can be drawn between the observations made in mouse models and in human trials regarding the potential impact of genotype, age and individual microbiota on probiotic effects. Understanding the influence of these factors and taking them in consideration during clinical trial design and data analysis would facilitate the development of improved probiotic interventions and strengthen the evidence for probiotic use in prevention and treatment of human disease.

Similarly to mouse studies, the influence of the human genotype has also been suggested to play an important role in the outcomes of probiotic interventions, including those performed in the context of allergic disease. For example, administration of a combined *Escherichia coli* Symbio and *E. faecalis* Symbio bacterial lysate can more effectively prevent eczema in children with single paternal heredity for atopy (Box 1) (Lau et al., 2012). Also, supplementation of *L. rhamnosus* HN001 and *Bifidobacterium* *infantis* subsp. *lactis* HN019 could alleviate childhood eczema susceptibility conferred by the presence of certain single nucleotide polymorphisms in the TLR genes in children (Marlow et al., 2015). Individual genetic variation has also been linked to differences in the composition of the microbiome, which further underlines the importance of host genetics in shaping microbe-host interactions (Blekhman et al., 2015; Knights et al., 2014). An association has previously been demonstrated between the count of specific risk alleles for inflammatory bowel disease in the host (e.g. *NOD2*) and an increased relative abundance of *Enterobacteriaceae* in the intestinal microbiome (Knights et al., 2014). These findings suggest that individual genetic differences and predisposition towards inflammatory diseases should be taken into consideration when assessing the effects of probiotics in a clinical setting.

The age of the host can likewise influence probiotic study outcomes in humans. In fact, the perinatal period was suggested to represent a window of opportunity for effective probiotic intervention in allergic disease in humans (Torow and Hornef, 2017). As such, meta-analyses of randomized controlled trials involving combined perinatal administration of probiotics to both pregnant mothers and newborns provide the strongest evidence of eczema prevention in children during the first 2 years of age (Pelucchi et al., 2012).

Both the age of the individual during probiotic administration and the influence of their individual microbiota have been suggested to play a role in human trials. For instance, administration of *L. rhamnosus* GG in combination with *L. rhamnosus* LC705, *B. breve* Bb99, *Proprionibacterium freudenreichii* ssp. shermani JS and prebiotic galactooligosaccharides to pregnant mothers, and subsequently to the infants, reduced eczema in children at 2 years of age (Kuitunen et al., 2009; Kukkonen et al., 2007). However, a reduction in IgE-associated allergies was only observed in infants delivered by caesarean section, which are known to harbour different microbiota in various niches compared with vaginally born children (Kuitunen et al., 2009). Although the important influence of human gut microbiota in carcinogenesis and response to antitumor therapy has recently been described in detail (Roy and Trinchieri, 2017), its impact in the context of probiotic treatment of allergic disease remains to be elucidated. It is conceivable that future probiotic applications could benefit from study subject stratification and a personalized approach based on the individual characteristics of the patient, including gene allele variation and microbiome composition. Additional well-designed studies and supportive *in vivo* and *in vitro* research are thus required to promote detailed selection of probiotic strains for prevention and treatment of allergic disease.

## Conclusion

In conclusion, the outcome of studies regarding the influence of probiotic interventions on various immune system functions can be greatly influenced by host parameters, which should be taken into consideration, both in animal and human studies. An adequate design of animal experiments will result in an improved predictive value of probiotic interventions in future clinical studies, and a more accurate assessment and extrapolation of the immune mechanisms involved. It would therefore be advisable to conduct experimental probiotic interventions in animal models closely resembling the envisioned human target group in regard to the variety of host-related factors. For example, by including different sex and age groups during experimental design in animals, it would be possible to avoid a skewed view of probiotic effects in the general population. Furthermore, accounting for sources of variation stemming from experimental protocol designs as well as the host would greatly contribute to experimental reproducibility. Once the promising effects of a probiotic strain are thoroughly described in different settings, it is then possible to focus on the most effective approaches, such as those targeting the neonatal window of immune development.

When certain host-related parameters, such as host genetics or resident microbiota, cannot be standardized as a result of study design, thorough investigation of these factors in the study subjects with subsequent subject stratification for data analysis should be considered. We therefore believe that conflicting results obtained in probiotic clinical trials could largely be explained by a lack of attention to these potential sources of variation. As heterogeneity in clinical studies with probiotics considerably complicates subsequent meta-analysis, increased awareness of the influence of host-related parameters and their acknowledgement in the context of probiotic interventions can aid appropriate patient stratification and greatly contribute to knowledge-based probiotic applications in future trials.
